# Molecular allergy diagnosis is sensitive and avoids misdiagnosis in patients sensitized to seasonal allergens

**DOI:** 10.1002/clt2.12231

**Published:** 2023-03-13

**Authors:** Lukas Koch, Karin Laipold, Lisa Arzt‐Gradwohl, Eva Maria Sturm, Werner Aberer, Martina Aumayr, Wolfgang Hemmer, Urban Čerpes, Gunter J. Sturm

**Affiliations:** ^1^ Department of Dermatology and Venereology Medical University of Graz Graz Austria; ^2^ Otto‐Loewi Research Center for Vascular Biology, Immunology and Inflammation Division of Pharmacology Medical University of Graz Graz Austria; ^3^ MacroArray Diagnostics GmbH Vienna Austria; ^4^ Floridsdorf Allergy Center Vienna Austria; ^5^ Allergy Outpatient Clinic Reumannplatz Vienna Austria

**Keywords:** cross‐reactivity, molecular allergy diagnosis, pollen, seasonal aeroallergens, sensitivity

## Abstract

**Background:**

The specificity of extract‐based pollen allergy diagnosis is decreased due to cross‐reactivity via cross‐reactive carbohydrate determinants (CCDs) or panallergens such as profilins or polcalcins. This study aimed to explore the prevalence of sensitization to seasonal extracts, CCDs, profilin and polcalcin and investigate the sensitivity and specificity of seasonal molecular allergy diagnosis (MAD) using commercially available test methods.

**Methods:**

2948 patients were screened for specific immunoglobulin E to ash, birch, mugwort, ragweed and timothy grass pollen extracts and grouped according to the number of positive tests (1–5). 100 patients from each group and a control group were randomly selected to calculate the prevalence of CCD and panallergen sensitization. With 742 patients, sensitivity and specificity of MAD (Alt a 1, Fra/Ole e 1, Bet v 1, Phl p 1, Art v 1, and Amb a 1) was determined.

**Results:**

1627 patients (55.2%) were positive to at least one, and 1002 patients (34.0%) were positive to multiple of the five pollen allergens investigated; 18.5% of the pollen‐sensitized patients had sensitization to CCDs or panallergens. Specifically, sensitization to CCDs, profilins, and polcalcins was observed in 8.7%, 10.9%, and 2.9% of these patients, respectively. The sensitivity of MAD was high, with sensitivities between 96.2% and 100% using ImmunoCAP and 91.5% and 100% using ALEX^2^. Specificity was 100% for both assays.

**Conclusions:**

Due to cross‐reactivity, about one‐fifth of pollen‐sensitized patients is at risk of misdiagnosis. However, MAD is sensitive, specific and helps to avoid misdiagnosis and select primary allergen sources for immunotherapy.

## 1 INTRODUCTION

Allergic rhinoconjunctivitis with or without asthma is one of the most frequent allergic diseases, affecting about 20% of the general population.^1^ Allergen immunotherapy is the only available treatment that targets the underlying pathophysiology and has a potential long‐term effect on reducing allergic symptoms. Selecting the appropriate (major) allergens for immunotherapy is crucial to achieve optimal effectiveness. Currently, prick testing and extract‐based specific immunoglobulin E (sIgE) determination are still the mainstays in diagnosing respiratory allergy. However, it has been known for years that cross‐reactive carbohydrate determinants (CCDs) or panallergens such as profilins or polcalcins can hamper or confound test results with native extracts by decreasing diagnostic specificity.

Sensitization to CCDs, profilins and polcalcins has been reported in about 22%–35%,^2^, ^3^, ^4^ 13%–50%,^5^, ^6^, ^7^, ^8^ and 8%–10%,^4^, ^5^, ^9^ respectively, with considerable variability depending on geographical regions and patient populations investigated. Nevertheless, the significant risk of misdiagnosis when using extract‐based diagnostic tests may have been underestimated because determining a wide range of molecular allergens or blocking of sIgE to CCDs was not routinely performed.

The benefit of molecular allergology and its impact on specific allergy diagnosis and therapy selection have been reported in several studies.^10^, ^11^, ^12^, ^13^ Previous sensitization rates to molecular marker allergens reported were as high as 80.7%–98.4% for Alt a 1,^14^, ^15^, ^16^, ^17^ 90%–100% for Amb a 1,^18^, ^19^, ^20^, ^21^, ^22^ >70%–>95% for Art v 1,^22^, ^23^, ^24^, ^25^, ^26^ 88.5%–97% for Bet v 1,^27^, ^28^, ^29^, ^30^ 97.7% for Fra e 1^31^ and 87.6%–88% for Ole e 1,^32^, ^33^ 74.8%–98% for Phl p 1,^34^, ^35^, ^36^, ^37^, ^38^, ^39^ and 27.2%–80% for Phl p 5.^8^, ^34^, ^35^, ^36^, ^37^, ^40^ However, most of these studies were based on experimental methods not routinely used or available such as immunoblotting, ELISA, or prick testing with molecular allergens.

This study had two goals. First, we aimed to determine the prevalence of multiple pollen sensitizations and of cross‐reactivity (via sensitization to CCDs, profilin, and polcalcin) in patients referred to allergy diagnostics and in patients with pollen sensitization. Second, we evaluated the sensitivity and specificity of molecular allergy diagnosis (MAD) with major seasonal marker allergens using the commercially available singleplex platform ImmunoCAP (Thermo Fisher Scientific, Waltham, MA, USA) and the multiplex array ALEX^2^ (Allergy Explorer version 2, MacroArray Diagnostics, Vienna, Austria).

## 2 METHODS

**FIGURE 1:**
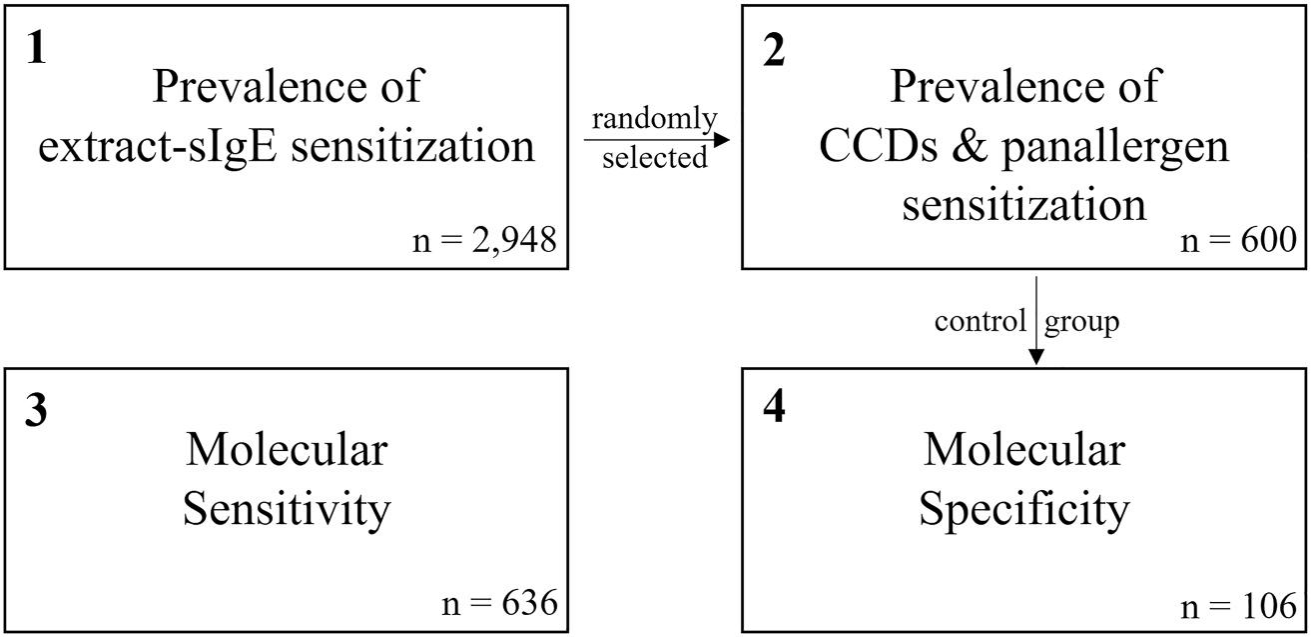
Flow‐chart of the four analyses performed. In total, clinical data and test results of 3.590 patients were investigated. Patients of analysis 2 were randomly selected from analysis 1 (6 × 100 patients with 0–5 pollen sensitizations). The control group from analysis 2 (100 patients without pollen allergy) and six additional patients were used for analysis 4.

For this retrospective study, clinical data and extract and prick test results of 3590 patients were investigated using four analyses (Figure 1). None of the patients in any of the analyses currently or previously received allergen‐specific immunotherapies.

### 2.1 Analysis 1. Prevalence of pollen sensitization

The frequency of (multiple) pollen sensitization was investigated in 2948 patients with rhinoconjunctivitis and/or bronchial asthma referred for allergy diagnostics between January 1 and December 31, 2020. Subjects were screened for pollen allergy using the determination of sIgE against five pollen extracts (ash, birch, timothy grass, mugwort, and ragweed) by ImmunoCAP. We classified subjects according to their number of sensitizations; patients showing sIgE to one to five different pollen extracts were grouped accordingly. In addition, because *Alternaria* is another important seasonal allergen in our region, prevalence of sIgE sensitization to *Alternaria* was investigated in all 2948 patients.

### 2.2 Analysis 2. Sensitization to CCDs and pollen panallergens

One hundred persons from each of the five groups mentioned above and a control group of 100 non‐allergic subjects were randomly selected. All controls had negative sIgE to the investigated six seasonal allergen extracts and *Dermatophagoides pteronyssinus* in the ImmunoCAP system and negative prick tests to 13 aeroallergens (pollen, pets, mites and moulds). In these 600 sera, the markers MUXF3 (ImmunoCAP), Phl p 7 (ALEX^2^), and Phl p 12 (ALEX^2^) were determined to detect the frequency of sensitization to CCDs, profilin, and polcalcin, respectively.

### 2.3 Analysis 3. Sensitivity of major marker allergens

To calculate the sensitivity of the molecular marker allergens of these five pollen species (Fra/Ole e 1, Bet v 1, Phl p 1, Art v 1, and Amb a 1) and *Alternaria* (Alt a 1), 636 patients visiting the outpatient clinic between 2013 and 2020 were included. All these patients had positive sIgE and prick tests to the respective extract and reported seasonal respiratory symptoms limited to the pollination period of the particular allergen. 106 mono‐sensitized patients were included for each allergen source, except for mugwort and ragweed. Cross‐reactivity was common in patients sensitized to mugwort and ragweed using extracts due to shared allergens; therefore, 25% of the 106 patients included in each of these two groups were serologically double‐sensitized.

### 2.4 Analysis 4. Specificity of major marker allergens

Specificity of Fra/Ole e 1, Bet v 1, Phl p 1, Art v 1, Amb a 1, and Alt a 1 was tested in 106 non‐allergic subjects (the 100 controls of analysis 2 and six additional subjects). As mentioned above, prick tests to 13 aeroallergens and sIgE to seven extracts in the ImmunoCAP were negative.

### 2.5 Skin tests

Skin prick tests were performed with extracts from ALK‐Abelló, Hørsholm, Denmark. Test results were considered positive in a wheal larger than 3 mm in diameter and erythema.

### 2.6 Singleplex sIgE testing

Specific IgE antibody levels in the patients' sera were measured using the ImmunoCAP 1000 platform. Specific IgE levels were expressed in kilo units per litre (kU/l), and sIgE values ≥0.35 kU/l were considered positive. For statistical analysis, levels >100 kU/l were rated as 100 kU/l.

### 2.7 Multiplex sIgE testing

The multiplex test system ALEX^2^ was performed according to the manufacturer's instructions. Specific IgE levels were expressed in kU/l, and sIgE values ≥0.30 kU/l were considered positive.

### 2.8 Sample size calculation & statistical analyses

Sample size calculation was performed using the statistical software R (version 3.6.1; The R Foundation, Vienna, Austria). Sensitivities between 0.96 and 0.98 were expected based on the sensitivity rates mentioned in the introduction. A sample size of 100 with an expected sensitivity of 0.96 would have resulted in an exact, two‐sided 95% confidence interval (CI) of 0.90 – 0.99 and with an expected sensitivity of 0.98 in a 95% CI of 0.93 – 1.00. Therefore, with an assumed drop‐out rate of 5%, 106 patients in each of the respective groups were required to show that the sensitivity is indeed above 90%.

For statistical analyses, McNemar's test, Wilcoxon signed‐rank test, and Spearman's rank correlation were performed using IBM SPSS Statistics 27 (IBM, Somers, USA). Graphs were generated using GraphPad Prism 9.0 (GraphPad Software, Inc., La Jolla, USA). The level of significance was set at 0.05.

Approval of the ethics committee of the Medical University of Graz for this study is available under approval no. 26–398 ex 16/17.

## 3 RESULTS

Demographical and clinical description of the four study populations (analysis 1: prevalence of (multiple) pollen sensitizations; analysis 2: sensitization to CCDs, profilins, and polcalcins; analysis 3: sensitivity of MAD; analysis 4: specificity of MAD) are shown in Table 1.

**TABLE 1:** Demographic data of the study populations. Asthma and bronchitis are defined by patients reporting chronic cough with and without an established diagnosis of asthma by a lung specialist.

	Analysis 1	Analysis 2	Analysis 3	Analysis 4
	Pollen sensitizations (*n* = 2948)	CCDs & panallergens (*n* = 600)	molecular sensitivity (*n* = 636)>	Molecular specificity (*n* = 106)
Sex				
Females	52.9%	50.2%	56.3%	67.9%
Age [years]				
Median	29	28	32	36
Interquartile ranges	17‐43	16‐41	20‐43	25‐54
Frequency of symptoms				
Rhinitis	79.5%	84.5%	93.7%	61.3%
Conjunctivitis	32.1%	50.0%	63.8%	26.4%
Bronchitis	20.1%	15.5%	16.8%	23.6%
Asthma	8.8%	8.8%	5.2%	7.5%

### 3.1 Prevalence of pollen sensitization

1660 (56.3%) of the 2948 patients were extract sIgE positive to at least one of the six seasonal allergen sources investigated, and 1627 (55.2%) were positive to at least one of the five pollen allergens. Multiple sensitizations to pollen were found in 34.0% of the overall study population. In the pollen‐sensitized patients, sensitization to all five pollens was found in 22.4%; 6.7% had four sensitizations, 12.8% had three, 19.8% had two, and 38.4% of the patients were positive to only one pollen source. Many sensitizations were associated with low levels of sIgE (16.9% of all positive pollen sIgE results in analysis 1 were below 0.7 kU/l), and considering cut‐offs above 0.7 or 3.5 kU/l, only 15.4% or 4.5% of the patients were positive to all five pollen species investigated (compared to 22.4% without cut‐off).

### 3.2 Correlation of sIgE and prick test results

Specific IgE and prick test results of the 2948 patients are shown in Table 2. It was remarkable that sIgE negative/prick positive patients were rarely seen for all allergens, but sIgE positive/prick negative patients were frequently observed for pollen allergens but not for *Alternaria*. On average, 13.3% of the 14740 pollen extract determinations had negative corresponding prick test results, compared to 2.9% of the *Alternaria* extract determinations. The difference of 10.4% may be explained by anti‐CCD IgE, reacting with pollen but not with *Alternaria* extract.

**TABLE 2:** Correlation of sIgE results and prick test results of 2948 patients. Patients were classified into four groups: sIgE extract positive, prick positive (“true‐positive results”); sIgE extract negative, prick negative (“true‐negative results”); sIgE extract negative, prick positive (“false‐negative sIgE or false‐positive prick test”); and sIgE extract positive, prick negative (“false‐positive sIgE or false‐negative prick test”). Pollen (mean) states the average percentages of all pollen investigated.

Allergen	sIgE +, prick +	sIgE −, prick −	sIgE −, prick +	sIgE +, prick −
Ash	9.2%	70.8%	1.2%	18.9%
Birch	20.0%	68.2%	1.7%	10.1%
Timothy grass	36.3%	54.0%	1.9%	7.7%
Mugwort	5.0%	81.2%	1.2%	12.6%
Ragweed	3.7%	78.8%	0.3%	17.2%
Pollen (mean)	14.8%	70.6%	1.3%	13.3%
*Alternaria*	4.6%	91.8%	0.8%	2.9%

### 3.3 Sensitizations to CCDs and pollen panallergens

Patients were grouped according to their number of pollen sensitizations (0–5), with one hundred randomly selected patients in each group. In these 600 sera (500 from patients with and 100 from controls without pollen allergy), MUXF3, Phl p 7, and Phl p 12 as markers for CCDs, polcalcins, and profilins, respectively, were determined (Table 3). As expected, sensitizations to CCDs and panallergens were most frequently observed in patients with positive results to all five pollen species; in this group, 73% were sensitized to CCDs or any panallergen, and 17% even showed sIgE to two panallergens or one panallergen and CCDs. In the group with four positive pollen extracts, pan‐sensitization was still relevant and observed in 17% of the patients, whereas this was rarely seen from group three sensitizations downwards. None of the 500 pollen allergic patients showed sIgE to all, CCDs and both panallergens. After extrapolating from 500 to 1627 patients with pollen sensitizations, up to 18.5% could react to at least one panallergen or CCDs. Specifically, sIgE to CCDs, profilins, and polcalcins could be expected in 8.7%, 10.9%, and 2.9%. None of the 100 patients in the control group did show any reactivity to CCDs, profilins, or polcalcins. After extrapolating from 600 to 2948 patients, up to 10.2% of all patients referred to our outpatient clinic for allergy diagnostics due to respiratory symptoms could be sensitized to at least one panallergen or CCDs (in detail, 4.8% to CCDs, 6.0% to profilins, and 1.6% to polcalcins).

**TABLE 3:** Reactivity to CCDs and the panallergens profilin and polcalcin. Patients were put into groups 0–5 according to the number of positive pollen extracts (ash, birch, timothy grass, mugwort, ragweed) with *n* = 100 for each group. In the control group (0), all pollen extracts were negative. Pan‐sensitizations: Coincidence of CCDs, profilin, and polcalcin sensitization. Total percentages were extrapolated for the overall study population (*n* = 2948).

Number of positive pollen extracts	CCD (MUXF3)	Profilin (Phl p 12)	Polcalcin (Phl p 7)	Pan‐sensitizations			
				1	2	3	1‐3
0	0.0%	0.0%	0.0%	0.0%	0.0%	0.0%	0.0%
1	0.0%	0.0%	1.0%	1.0%	0.0%	0.0%	1.0%
2	0.0%	0.0%	0.0%	0.0%	0.0%	0.0%	0.0%
3	1.0%	1.0%	3.0%	5.0%	0.0%	0.0%	5.0%
4	7.0%	10.0%	1.0%	16.0%	1.0%	0.0%	17.0%
5	36.0%	45.0%	9.0%	56.0%	17.0%	0.0%	73.0%
Total	4.8%	6.0%	1.6%	8.1%	2.1%	0.0%	10.2%

We did not observe any sensitization to profilins or polcalcins without concomitant reactivity to at least one major pollen allergen in any of the 600 investigated sera. The primary mono‐sensitizer in sera with profilin or polcalcin sensitization was timothy grass, birch, or ash pollen in 18.2%, 3%, and 3%, respectively, whereas multiple genuine pollen sensitizations were detected in 75.8%. Moreover, only three of the 600 sera showed CCD reactivity without any molecular sensitization to pollen. Presumably, their anti‐CCD IgE was due to insect venom sensitization (all three patients had reactivity to Ves v 5), and these antibodies were able to elicit false‐positive pollen extract sIgE results.

### 3.4 Correlation of sIgE to extracts and major molecular marker allergens

In the 500 pollen‐allergic sera mentioned above, only 985 (65.7%) of the 1500 positive extract results had positive corresponding major molecular allergens, which means that 34.3% of the positive extract results were not due to sensitization to the respective molecular marker allergens. The more extracts were positive, the lower was the correlation between extracts and molecular marker allergens: in the one‐to five‐fold (extract) pollen‐positive sera, 92.0%, 85.0%, 79.7%, 59.3%, and 49.4% of the reactive extracts had positive corresponding marker allergens. Correlation differed between the five pollen species investigated: 91.4%, 77.7%, 74.0%, 29.6%, and 21.3% of the timothy grass, birch, ash, mugwort, and ragweed pollen extract positive patients had positive corresponding molecular allergens, respectively.

### 3.5 Sensitivity of MAD

The sensitivity of major molecular allergens was investigated in 106 genuinely sensitized subjects per allergen source. Sensitivity was high, with percentages between 91.5% and 100% using ALEX^2^ and 96.2% and 100% using ImmunoCAP. In addition, the molecular sensitivity correlated to pollen extract sIgE levels and increased with higher sIgE to the extracts (Table 4).

**TABLE 4:** Sensitivity of MAD using ImmunoCAP and ALEX^2^. The sensitivity of both molecular assays increased with higher sIgE levels to pollen extracts. For the molecular diagnosis of ash pollen allergy, Ole e 1 was used with ImmunoCAP as no Fra e 1 was available. All *p*‐values listed are direct comparisons to extract‐based singleplex diagnosis using ImmunoCAP. No *p*‐values could be calculated for 100% sensitivity due to constant values.

		**ImmunoCAP**	*p*‐value	ALEX^2^	*p*‐value
Extracts ≥0.35kU/l					
*Alternaria*	Alt a 1 (*n* = 106)	100%		100%	
Ragweed	Amb a 1 (*n* = 106)	97.2%	0.250	97.2%	0.250
Mugwort	Art v 1 (*n* = 106)	96.2%	0.125	91.5%	0.004
Birch	Bet v 1 (*n* = 106)	100%		100%	
Ash	Fra e 1 (*n* = 106)			98.1%	0.500
	Ole e 1 (*n* = 106)	98.1%	0.500		
Timothy grass	Phl p 1 (*n* = 106)	97.2%	0.250	98.1%	0.500
	Phl p 1 + 5 (*n* = 106)	100%		100%	
Extracts ≥0.7 kU/l					
*Alternaria*	Alt a 1 (*n* = 106)	100%		100%	
Ragweed	Amb a 1 (*n* = 103)	98.1%	0.500	99.0%	1.000
Mugwort	Art v 1 (*n* = 89)	96.6%	0.250	94.4%	0.063
Birch	Bet v 1 (*n* = 102)	100%		100%	
Ash	Fra e 1 (*n* = 104)			98.1%	0.500
	Ole e 1 (*n* = 104)	98.1%	0.500		
Timothy grass	Phl p 1 (*n* = 106)	97.2%	0.250	98.1%	0.500
	Phl p 1 + 5 (*n* = 106)	100%		100%	
Extracts ≥3.5 kU/l					
*Alternaria*	Alt a 1 (*n* = 75)	100%		100%	
Ragweed	Amb a 1 (*n* = 84)	100%		98.8%	1.000
Mugwort	Art v 1 (*n* = 23)	100%		95.7%	1.000
Birch	Bet v 1 (*n* = 89)	100%		100%	
Ash	Fra e 1 (*n* = 74)			100%	
	Ole e 1 (*n* = 74)	100%			
Timothy grass	Phl p 1 (*n* = 96)	99.0%	1.000	99.0%	1.000
	Phl p 1 + 5 (*n* = 96)	100%		100%	

The overall sensitivity of Art v 1 was lower using ALEX^2^ compared to the ImmunoCAP (91.5% vs. 96.2%). Determination of Art v 1 using ImmunoCAP was comparable to extract‐based diagnosis (*p* = 0.125), whereas determination with ALEX^2^ was not (*p* = 0.004). However, if sIgE to the mugwort extract available on ALEX^2^ was included, the overall sensitivity of the ALEX^2^ increased to 94.3%. In patients with mugwort sIgE levels greater than 0.7 kU/l, Art v 1 performed statistically equally to extract‐based diagnosis in both test systems. Phl p 1 showed a high sensitivity in both test systems and was statistically equal to extract‐based diagnosis (97.2% ImmunoCAP vs. 98.1% ALEX^2^); however, adding sIgE determination to Phl p 5 increased sensitivity to 100% in both systems.

Interestingly, the sensitivity of the surrogate marker for ash pollen allergy using ImmunoCAP, Ole e 1, was 98.1% and identical to that of Fra e 1 on ALEX^2^. Alt a 1 of Alternaria and Bet v 1 of birch pollen showed 100% sensitivity with both test methods.

### 3.6 Specificity of MAD

The major molecular allergens Alt a 1, Amb a 1, Art v 1, Bet v 1, Fra/Ole e 1 and Phl p 1 were tested in 106 non‐allergic controls. None of these 106 subjects was positive for any of these molecular marker allergens resulting in a 100% specificity for all allergens.

### 3.7 Correlation of the molecular test results

**FIGURE 2:**
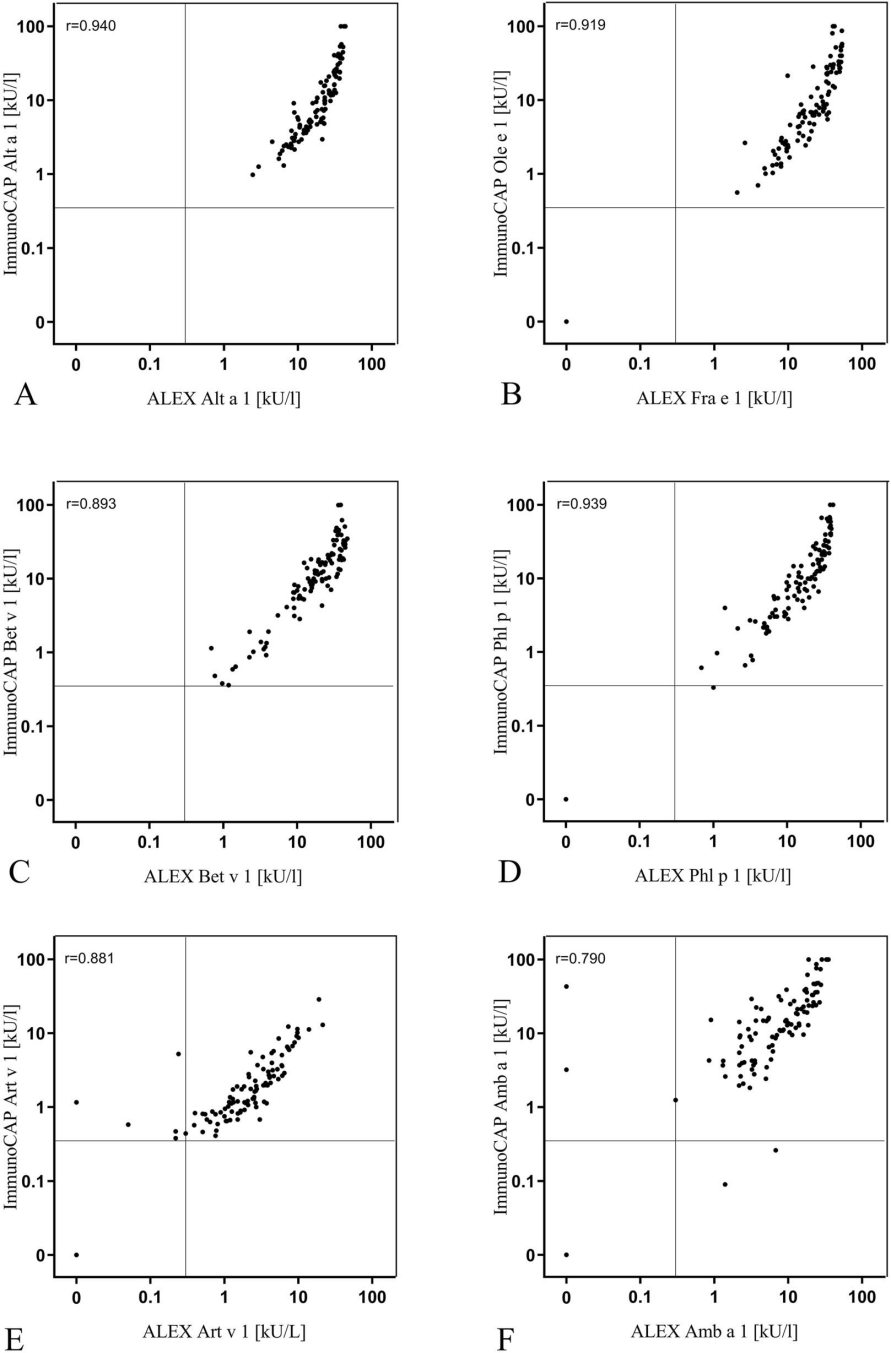
Correlation of the molecular allergy test systems. Scatter plots and Spearman's rho results of the molecular allergens of *Alternaria* (Alt a 1), ash (Fra e 1 and Ole e 1), birch (Bet v 1), timothy grass (Phl p 1), mugwort (Art v 1) and ragweed (Amb a 1) determined using ImmunoCAP and ALEX^2^ are shown.

MAD on both platforms correlated strongly with Spearman's rho ranging between 0.790 and 0.940 (Figure 2). However, mean sIgE values to the major allergens differed significantly between ALEX^2^ and ImmunoCAP (*p* < 0.001 for all six allergens). For example, mean sIgE to Amb a 1 was 10.9 and 22.4 kU/l, respectively, and therefore lower in ALEX^2^. However, mean sIgE to all other allergens was higher in ALEX^2^ compared to ImmunoCAP: 21.3 and 15.3 kU/l for Alt a 1, 3.4 and 2.9 kU/l for Art v 1, 21.9 and 16.5 kU/l for Bet v 1, 25.2 and 16.0 kU/l for Fra e 1 and Ole e 1, and 20.0 and 18.6 kU/l for Phl p 1, respectively.

## 4 DISCUSSION

Extract‐based prick testing and sIgE determination are still the mainstays in the diagnosis of respiratory allergy. Unfortunately, the specificity of these tests is impaired because of cross‐reactivity to CCDs or panallergens such as profilin or polcalcin. For years, it has been known that CCDs and panallergens such as profilin and polcalcin can distort diagnosis. However, the considerable risk of misdiagnosis when using extract‐based diagnostic tests may have been underestimated because the determination of a wide range of molecular allergens or blocking of sIgE to CCDs were not routinely performed. Costs or the (un)availability of molecular singleplex and multiplex sIgE determinations may have prevented broad application. About one‐fifth (18.5%) of our pollen‐sensitized patients and about one‐tenth (10.2%) of all our patients referred to allergy diagnosis revealed cross‐reactivity. The rate of sensitization to CCDs and panallergens observed in our study is in agreement with previously published data.^2^, ^3^, ^4^, ^5^, ^6^, ^7^, ^9^ Consequently, in daily routine, many patients are at risk of being incorrectly diagnosed and prescribed a potentially inaccurate immunotherapy.

There are two options to handle the risk of cross‐reactivity when using sIgE‐based in vitro pollen allergy diagnosis: the determination of a predefined pollen extract panel in every patient or the use of molecular pollen allergy diagnosis.

First, if molecular allergology is not available, a representative predefined pollen extract panel (in Central Europe: ash, birch, timothy grass, mugwort, and ragweed) should be determined in all patients. Certainly, in other European regions the panel should be modified according to local botanical situations, especially in the weed population. Sensitization to multiple pollen can be expected in about one‐third of the patients suffering from rhinoconjunctivitis with or without bronchial asthma and is a warning sign for cross‐reactivity. In our population, 73% of the patients with five pollen sensitizations and 17% with four pollen sensitizations showed any type of cross‐reactivity. Therefore, corresponding major molecular marker allergens could not confirm 50.6% and 40.7% of these patients' positive pollen extract results, indicating that the extract‐based results were without, or only of minor, clinical significance. Conversely, if only three or fewer out of these five pollens were positive, the risk of cross‐reactivity was minimal (5%) or negligible (≤1%). This approach helped to recognize cross‐reactivity but still requires careful interpretation of the test results.

The second approach to minimize the risk of cross‐reactivity is molecular allergology. In a recent review article by Barber et al, the benefit of molecular allergology and its impact on specific allergy diagnosis and therapy has been shown.^10^ In addition, multiple studies have demonstrated that the use of molecular allergology leads to different therapeutic decisions compared to extract‐based testing.^11^, ^12^, ^13^ The reported sensitivities of molecular pollen marker allergens are promising;^14^, ^15^, ^16^, ^17^, ^18^, ^19^, ^20^, ^21^, ^22^, ^23^, ^24^, ^25^, ^26^, ^27^, ^28^, ^29^, ^30^, ^31^, ^32^, ^33^, ^34^, ^35^, ^36^, ^37^, ^38^, ^39^ however, their transferability to routine diagnostics was questionable, because percentages varied considerably and many of the studies were performed with tests which are not routinely available such as immunoblotting, ELISA or prick testing with molecular allergens.

Our study indicates that the determination of single molecular marker allergens using commercially available test methods is highly sensitive and specific to diagnose the six most frequent seasonal inhalant allergens in Central Europe. For most allergen sources, only one molecular marker allergen is sufficient. Nevertheless, in grass pollen allergy, more than one could be relevant. For example, we reported earlier that Phl p 1 was enough to diagnose timothy grass pollen allergy with a sensitivity of 98%.^39^ Our current study confirms this finding; however, additional testing of Phl p 5 could increase sensitivity to 100%. Furthermore, mono‐sensitization to Phl p 2 or Phl p 4 may be relevant for some patients as it was rarely observed in some geographical regions.^41^ Only mugwort allergy could not be optimally diagnosed on the multiplex platform ALEX^2^. Overall sensitivity was 91.5% using Art v 1 and 94.3% using Art v 1 in combination with the mugwort extract; however, this may be explained as not being due to technical problems of the ALEX^2^ but the selection of our mugwort patients. Our patients with mono‐sensitization to mugwort usually had very low levels of sIgE, and the number of patients with sIgE levels <0.7 kU/L was clearly above average compared to all other allergen sources (Table 4). It is known that the sensitivity of multiplex systems, such as ALEX^2^, could be lower in patients with low sIgE levels due to higher limits of detection, higher coefficients of variation, and potential inhibition by antigen‐specific IgG.^42^ We previously showed that the higher the sIgE to allergen extracts, the better was the sensitivity of molecular allergy testing in diagnosing house dust mite allergy.^43^ The high rate of patients with low sIgE to mugwort may explain the observed lower sensitivity of Art v 1. However, because its sensitivity was equal to the extract‐based approach in patients with sIgE levels greater than 0.7 kU/l in both methods and because the major allergen content of mugwort allergen immunotherapies is adjusted to Art v 1, it is definitely suitable for diagnostic use.

The singleplex assay ImmunoCAP and the multiplex platform ALEX^2^ correlated strongly and produced almost the same results when the diagnosis was performed with major molecular allergens. Both methods are appropriate for routine diagnostics though neither system is perfect: drawbacks of the ImmunoCAP are the costs for a comprehensive MAD and, theoretically, interference of anti‐CCD antibodies with the cellulose used as a solid‐phase allergen carrier.^44^ On the other hand, multiplex platforms such as ALEX^2^ may have a lower sensitivity in patients with very low levels of sIgE. However, a broad application of MAD in pollen‐allergic patients with any method could enhance the quality of allergy diagnosis and, consequently, the effectiveness of allergen immunotherapy.

The main limitation of our study, besides its retrospective design, is that we have investigated a Central European study population. Therefore, additional allergens (e.g. Phl p 2 or 4) or allergen sources (e.g. olive tree, parietaria or plantain pollen) may be relevant in other distinct geographical regions, and the sensitization rates to CCDs or pan‐allergens may vary.

In summary, about one‐fifth (18.5%) of our pollen‐sensitized patients and about one‐tenth (10.2%) of all our patients referred to allergy diagnosis were affected by cross reactivity; therefore, cross‐reactivity is common and may lead to misdiagnosis and consequently to a 3‐year immunotherapy with inadequate allergen vaccines. In contrast, MAD determining Alt a 1, Amb a 1, Art v 1, Bet v 1, Fra/Ole e 1 and Phl p 1 with commercially available methods is highly sensitive and specific and helps to avoid misdiagnosis and select primary allergen sources for immunotherapy.

## AUTHOR CONTRIBUTIONS

**Lukas Koch**: Conceptualization (Equal); Data curation (Equal); Formal analysis (Equal); Funding acquisition (Equal); Investigation (Equal); Methodology (Equal); Project administration (Equal); Software (Equal); Supervision (Equal); Validation (Equal); Visualization (Equal); Writing – original draft (Equal); Writing – review & editing (Equal); **Karin Laipold**: Data curation (Equal); Investigation (Equal); Validation (Equal); Writing – review & editing (Equal); **Lisa Arzt‐Gradwohl**: Formal analysis (Equal); Investigation (Equal); Software (Equal); Validation (Equal); Visualization (Equal); Writing – review & editing (Equal). **Eva Maria Sturm**: Conceptualization (Equal); Investigation (Equal); Methodology (Equal); Project administration (Equal); Supervision (Equal); Writing – review & editing (Equal). **Werner Aberer**: Conceptualization (Equal); Methodology (Equal); Project administration (Equal); Supervision (Equal); Writing – review & editing (Equal). **Martina Aumayr**: Resources (Equal); Validation (Equal); Writing – review & editing (Equal). **Wolfgang Hemmer**: Data curation (Equal); Validation (Equal); Writing – review & editing (Equal). **Urban Čerpes**: Data curation (Equal); Investigation (Equal); Validation (Equal); Writing – review & editing (Equal). **Gunter J. Sturm**: Conceptualization (Equal); Data curation (Equal); Formal analysis (Equal); Funding acquisition (Equal); Investigation (Equal); Methodology (Equal); Project administration (Equal); Resources (Equal); Software (Equal); Supervision (Equal); Validation (Equal); Visualization (Equal); Writing – original draft (Equal); Writing – review & editing (Equal).

## CONFLICTS OF INTEREST STATEMENT

Martina Aumayr is an employee of MacroArray Diagnostics. All other authors declare no conflict of interest in relation to this work.

## Data Availability

The data that support the findings of this study are available on request from the corresponding author. The data are not publicly available due to privacy or ethical restrictions.
